# One-year clinical outcome after full-endoscopic interlaminar lumbar discectomy for isthmic lumbar spondylolisthesis: Two case reports

**DOI:** 10.1097/MD.0000000000026385

**Published:** 2021-06-25

**Authors:** Takeshi Kaneko, Yuichi Takano, Hirohiko Inanami

**Affiliations:** Inanami Spine and Joint Hospital, Higashishinagawa, Shinagawa-ku, Tokyo, Japan.

**Keywords:** 1-year clinical outcome, FESS, isthmic lumbar spondylolisthesis, lumbar disc herniation

## Abstract

**Rationale:**

For isthmic lumbar spondylolisthesis (ILS) associated with the removal of herniation, it remains challenging to perform less invasive and minimally disruptive procedures. Good results could potentially be obtained by further preserving the posterior elements in full-endoscopic lumbar discectomy (FESS), which is less invasive than microenscopic surgery (MES).

**Patient concerns:**

One patient complained of left leg pain, and another patient complained of right leg pain and low back pain.

**Diagnoses:**

Two patients with ILS and Meyerding Grade 1 lumbar spondylolisthesis.

**Interventions:**

We performed a full-endoscopic lumbar discectomy via the interlaminar space (FESS-IL) for L5/S1 lumbar disc herniation (LDH) accompanied by isthmic lumbar spondylolisthesis. FESS-IL was performed in 2 patients with radiculopathy caused by different types of LDH using a full endoscopic system with a 4.1 mm working channel and 6.9 mm outer diameter. A 3.5-mm diameter high-speed drill was used in one patient for an upward-migrated LDH in the inner-rim of the infravertebral border. The other patient underwent minimal resection without bone resection.

**Outcomes:**

The one-year clinical outcome included confirmation of pain relief and evacuation of migrated LDH on magnetic resonance imaging in all patients. There was no progression of slippage on radiography. The mean operative time was 82 min, and no complication was observed. The one-year clinical outcome demonstrated sufficient pain relief.

**Lessons: The 1-y:**

ear postoperative outcome showed improvement. We believe that FESS-IL is a viable alternative operative approach for LDH for ILS.

## 1 Introduction

Full-endoscopic lumbar discectomy (FESS) with a 4.1 mm working channel is one of the minimally invasive operative procedures for the treatment of lumbar disc herniation (LDH).^[1,2]^ For isthmic lumbar spondylolisthesis (ILS) associated with the removal of herniation, it remains challenging to perform less invasive and minimally disruptive procedures even with FESS. Good results with microendoscopic discectomy (MED) have been reported for lumbar spondylolysis.^[3]^ We believe that in FESS, which is less invasive, good results could be obtained by further preserving the posterior elements. In this report, we performed FESS on cases with spondylolysis. We report two cases and present their clinical outcomes including the preoperative and 1-year postoperative Oswestry Disability Index (ODI) and Roland–Morris Disability Questionnaire (RDQ-24). This study was approved by ethics committee of the Iwai Medical Foundation, and informed consent was obtained from patients for the purpose of publication.

## 2 Case presentation 1

**Figure 1:**
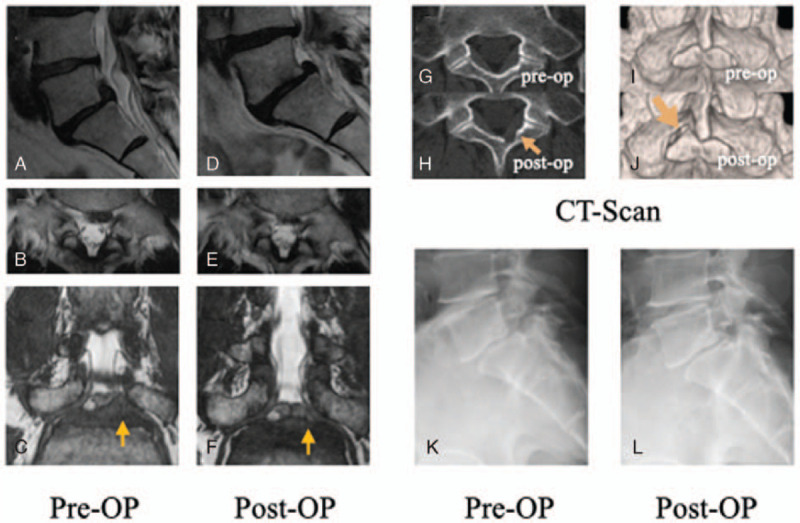
A 55-year-old man who had been suffering from radiculopathy of the L5 area with spondylolisthesis underwent a full endoscopic discectomy via the interlaminar space (FESS-IL) for L5/1. At 1 year after surgery, there was no sign of recurrence, herniation, or progression of slippage (K and L). Preoperative sagittal (A) axial (B) and coronal (C) images on T2-weighted magnetic resonance imaging (MRI), and axial and 3D image on computed tomography (CT) (G and I). Postoperative (1 year) sagittal (D) axial (E) and coronal (F) images on T2-weighted MRI. Axial and 3D-CT image on CT (H and J) shows only a small amount of drilling was performed on the inferior border of the lamina.

A 55-year-old man presented with left leg pain (L5 dermatomes) and low back pain that started 2 months before visiting our outpatient clinic. Although neurological examination revealed a positive straight-leg rising sign (SLR) at 50° on the left side, no apparent muscle weakness was observed. His symptoms were resistant to medical treatment (pregabalin 225 mg and tramadol). Radiographic imaging revealed ILS and Meyerding Grade 1 lumbar spondylolisthesis (Fig. 1K). Lumbar magnetic resonance imaging (MRI) revealed an upward-migrated LDH at the L 5/1 disc level and compressed L5 nerve root (Fig. 1A–C). Three-dimensional computed tomography (3D-CT) demonstrated a large L5/S1 interlaminar bone window (Fig. 1G, I). The patient opted for treatment other than fusion due to difficulties involving long hospital stays to care for their parents and potential restrictions in activities of daily living. Furthermore, in order to restore the facets and other structures, we hypothesized that the sequestrated nucleus could be extracted through the aforementioned bone window. Under general anesthesia, we performed FESS-IL after insertion to the inferior border of the lamina. As the working channel could not reach the intended apex of the hernia, a 3.5 mm high-speed drill (NSK-Nakanishi Japan, Tokyo, Japan) was used to carve out the medial border of the lamina. A Penfield probe was subsequently used to confirm that the apex of the hernia was reached. On the shoulder area of the left S1 nerve root, the endoscope was tilted toward the L5/1 disc space, and the sequestrated nucleus was pulled out. No drainage was applied. At 4 h postoperatively, the leg pain improved. The patient was discharged on POD1. Postoperative CT was performed below the incision of the inferior border alone (Fig. 1H, J). The 1-year postoperative MRI revealed complete disappearance of the superiorly-migrated LDH (Fig. 1D–F). The 1-year outcome was improved in all categories (Table 1). Radiography did not show any progression of slippage (Fig. 1L).

**Table 1:** Preoperative and 1 year postoperative clinical outcome of FESS-IL for a L5/S1 LDH accompanied by ILS.

Age	Gender	Operation time (min)	Pre-op ODI	Post-op ODI	Pre-op RDQ24	Post-op RDQ24	Pre-op NRS	Post-op NRS
55	Male	84	36	4	12	0	10	0
51	Female	80	18	2	7	0	8	0

NRS = Numerical Rating Scale, ODI = Oswestry Disability Index (ODI), Post-op = postoperative, Pre-op = preoperative, RDQ-24 = Roland–Morris Disability Questionnaire.

## 3 Case presentation 2

**Figure 2:**
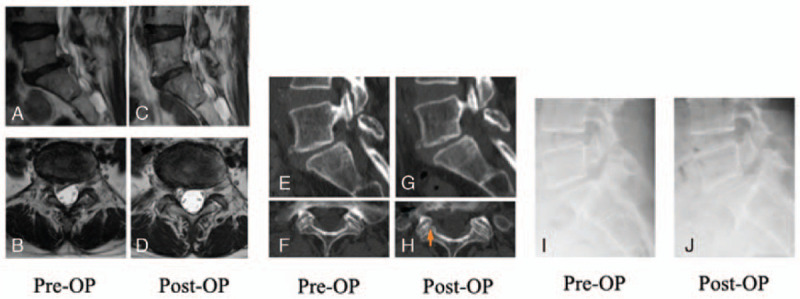
A 51-year-old female who had disc herniation at L5/1 and concomitant L5 spondylolisthesis underwent Full endoscopic discectomy via the interlaminar space (FESS-IL) for L5/1. At 1 year after surgery, there was no sign of recurrence of herniation or progression of spondylolisthesis (I and J). Preoperative sagittal (A) and axial (B) images on T2-weighted magnetic resonance imaging (MRI), and sagittal and axial images on computed tomography (CT) (E&F). Postoperative (1 year) sagittal (C) and axial (D) images on T2-weighted MRI, and sagittal and axial images on CT (G&H).

A 51-year-old female presented with right leg pain (S1 dermatomes) that started 2 months before visiting our outpatient clinic. The patient did not complain of low back pain. Neurological examination revealed a positive SLR at 30° on both sides with no apparent muscle weakness. Her symptoms were resistant to medical treatment (celecoxib 200 mg). Radiographic imaging revealed ILS and Meyerding Grade 1 lumbar spondylolisthesis (Fig. 2 E&I). The lumbar MRI revealed intracranial LDH at the L5/S1 disc level (Fig. 2 A&B). The pain was reproducible with a selective S1 nerve block. The main symptom was herniation at the same disc level, and surgery was performed. Under general anesthesia, an incision was made 14 mm lateral to the spinous process. The superior border of the S1 lamina was identified under imaging, and the medial side of the S1 superior articular process (SAP) was confirmed with a Penfield probe. The ligamentum flavum was then excised with forceps. The ligamentum flavum was split cephalocaudally with the Penfield probe, the ligamentum flavum was excised mediolaterally with forceps, and a part of SAP was excised with a Kerrison rongeur to confirm the route. The Penfield probe was inserted lateral to the route. A cannula was inserted to avoid the route, and the hernia was identified. The internal hernia was removed. He was able to walk at 4 h postoperatively, and his leg pain was alleviated. Radiographic imaging showed no progression of slippage (Fig. 2 J). The outcome at 1 year postoperatively showed improvement (Table 1) with no recurrence on MRI (Fig. 2 D&E), and the patient is actively involved in sports.

## 4 Discussion

The aim of this study was to relieve pain while keeping surgical intervention as minimally invasive as possible. In both cases, the ODI at 1 year after surgery was significantly improved. Improvement of lower back pain was also observed in RDQ24. In terms of ILS, FESS-IL was believed to be a viable alternative procedure. In Case 1, in addition to the lumbar spondylolisthesis at L5, a superior migration of hernia that compressed the L5 route was observed. A high-speed drill was used to perform as little osteotomy as possible to carve the medial border of the L5 lamina and remove the migrated herniation.

Some concerns remain on the progression of slippage when opting not to perform fusion surgery.^[4]^ However, in both cases, neither progression of slippage nor increased back pain were observed even at 1 year after surgery. Full-endoscopic surgery results in less posterior obstruction to the paravertebral muscle than conventional endoscopy. There are also reports that there was no mechanical change,^[5,6]^ suggesting that FESS is a suitable surgical option for spondylolisthesis. Furthermore, the transforaminal approach (TFA) at the L5S level is affected by the L5 being the longest intervertebral foramen^[7]^ and the high iliac crest, making the surgical exposure of the channel difficult to obtain freely. When approaching a severely displaced hernia, the superior articular process must be carved out in order to obtain an unrestricted working space.^[8,9]^ In cases similar to Case 1, many reports in the literature have recommended the IL approach.^[10,11]^ We also believe that a small amount of the medial border from the inferior border of the lamina could be removed with a drill to access the hernia; thus, the amount of osteotomy was smaller than that of TFA, and a sufficient degree of freedom could be obtained in terms of the working space. Based on the above, FESS-IL was selected as a surgical alternative. This is a short-term outcome at 1 year after surgery. Further clarification is needed for long-term clinical results. Treatment options tailored to various lifestyles are considered to be an important factor in a rapidly aging society. Since there are potential drawbacks for fusion surgery such as adjacent facet injury, we believe the addition of FESS-IL surgery as an alternative procedure is beneficial for broadening the choice of treatment.

## 5 Conclusions

We reported a case of lumbar spondylolisthesis complicated by hernia. The 1-year postoperative outcome showed improvement. In terms of ILS, the FESS-IL approach was a useful alternative procedure. Even in spondylolisthesis, fusion surgery may not always be necessary.

## Author contributions

**Conceptualization:** Takeshi kaneko.

**Data curation:** Takeshi kaneko.

**Formal analysis:** Takeshi kaneko.

**Investigation:** Takeshi kaneko.

**Methodology:** Takeshi kaneko.

**Project administration:** Takeshi kaneko.

**Resources:** Takeshi kaneko.

**Software:** Takeshi kaneko.

**Supervision:** Hirohiko Inanami.

**Validation:** Yuichi Takano.

**Writing – original draft:** Takeshi kaneko.

**Writing – review & editing:** Takeshi kaneko.
